# Cultural adaptation and psychometric assessment of the Persian version of the lumbar spine instability questionnaire

**DOI:** 10.1186/s13102-022-00486-2

**Published:** 2022-05-19

**Authors:** Noureddin Nakhostin Ansari, Zahra Abdollahzade, Soofia Naghdi, Mohammad Taghi Beigmohammadi, Mina Kashi-Alashti

**Affiliations:** 1 Sports Medicine Research Center, Neuroscience Institute, Tehran University of Medical Sciences, Tehran, Iran; 2 Research Center for War-Affected People, Tehran University of Medical Sciences, Tehran, Iran; 3 Department of Physiotherapy, School of Rehabilitation, Tehran University of Medical Sciences, Tehran, Iran; 4 Department of Anesthesiology and Intensive Care, Imam Khomeini Hospital Complex, Tehran University of Medical Sciences, Tehran, Iran

**Keywords:** Instability, Low back pain, Persian, Validity, Reliability, Questionnaire

## Abstract

**Background:**

The Lumbar Spine Instability Questionnaire (LSIQ) is a self-reported measure of clinical instability of the lumbar spine. This study aimed to translate and culturally adapt the LSIQ into Persian language (LSIQ-P) and to evaluate its reliability and validity in a sample of patients with chronic non-specific low back pain (LBP).

**Methods:**

In a cross-sectional study, the LSIQ was translated using guidelines. Participants with chronic non-specific LBP, aged ≥ 18 years old, answered an online survey consisting of LSIQ-P, the Persian Functional Rating Index (FRI), and the pain Numeric Rating Scale (NRS). Construct validity, internal consistency reliability, test–retest reliability, standard error of measurement (SEM), smallest detectable change (SDC), discriminant validity, and factor analysis were evaluated.

**Results:**

The LSIQ was successfully adapted into Persian. A sample of 100 participants with LBP and 100 healthy subjects completed the survey. Floor and ceiling effects were not observed. Cronbach’s alpha = 0.767 and ICC_agreement_ = 0.78 indicated good internal consistency and test–retest reliability. The SEM and SDC were 1.53 and 4.24, respectively. Construct validity of LSIQ-P was confirmed with significant correlation with Persian FRI (r = 0.44, *p* < 0.001) and pain NRS (r = 0.30, *p* = 0.003). An evidence of discriminant validity was demonstrated by significant difference in LSIQ-P total scores between the patients with LBP and healthy subjects, and between the patients with high total score ≥ 9 and those with low total score < 9 on the LSIQ-P. The LSIQ-P was found a multidimensional instrument with eight items appeared being redundant.

**Conclusions:**

The Persian LSIQ showed satisfactory metric characteristics of reliability and validity. Further studies are required to elucidate the internal structure of the LSIQ-P.

## Background

Low back pain (LBP) is a highly prevalent health condition affecting people from all age and gender groups [1]. LBP has been found as one of the leading causes of years lived with disability [2] and results in high healthcare costs and lost productivity [3, 4]. There are specific and non-specific forms of LBP. Non-specific LBP is identified if there is no known cause and pathology. Most of the patients with LBP are diagnosed as non-specific LBP. A recent study found a lifetime prevalence of non-specific LBP as being 62.6% [5]. However, patients with non-specific LBP should be considered with subgroups of patients who their outcomes may differ in response to interventions [6, 7]. Patients with lumbar spine instability (LSI) are a known subgroup of chronic LBP population [8]. Lumbar spine instability is prevalent in patients with LBP. A study found 57% of patients with LBP had radiographic instability [9]. Clinically, patients with LSI have lost the ability of their spine to maintain the movement patterns under physiological loads [10]. The identification of LBP patients with LSI using valid and reliable tools is essential to apply timely physical therapy interventions to prevent structural damages to spine.

Radiographic examination is an objective measure for identifying patients with LSI. However, access to the radiography equipment, time and cost to administer it, and radiation exposure limit its utility for routine use in the clinics [11]. There are numerous clinical tests for use in clinical practice to diagnose the LSI. However, a systematic review to evaluate the clinical tests found limitations in their diagnostic accuracy and validity [12]. Questionnaires are widely used in the clinics and research. Although numerous health related questionnaires have been developed to measure pain and dysfunction in patients with LBP, specific questionnaires particularly for LSI is scarce. Self-reported questionnaires have beneficial for use in the clinics. They do not require specific skills to administer, can be used as adjuncts for diagnosis, can help to measure the treatment effects, and are helpful where there is shortage of equipment.

It is important to diagnose patients with LSI in the clinics using useful instruments. Lumbar Spine Instability Questionnaire (LSIQ) is a measure developed as a self-reported instrument of clinical instability in patients with LBP [13–15]. The LSIQ has been previously used in the context of clinical investigations and showed acceptable metric characteristics with ability to predict the responses of LBP patients to motor control exercises [14, 15]. A study to evaluate the clinimetric characteristics of LSIQ in 107 patients with chronic non-specific LBP showed adequate test–retest reliability and one-dimensional construct validity [16]. The LSIQ has been culturally adapted into several languages of Thai [17, 18], Brazilian Portuguese [19], and Swedish [20]. There is no tool in Persian language to identify patients with LSI. Therefore, the aim of the present study was to culturally adapt the LSIQ into Persian language and evaluate the reliability and validity in patients with chronic non-specific LBP.

We in particular considered floor and ceiling effects, internal consistency reliability, test–retest reliability, absolute reliability measures, construct validity, and factor analysis.

## Methods

### Study design

The present study followed a cross-sectional design. The approval of study protocol was obtained from the Review Board, Sports Medicine Research Center, and the Ethics Committee of the Neuroscience Institute, Tehran University of Medical Sciences (Code: IR.TUMS.VCR.REC.1397.1032). The written informed consent was obtained from patients.

### Translation and cultural adaption

The standard methodology involving the forward and backward translation, expert panel review, and pilot-testing was followed as used previously [21, 22]. Briefly, translation into Persian language was performed by two bilingual individuals. Another two independent translators back translated the synthesized version into English. Expert panel reviewed the all documents and approved it for pilot testing. Expert panel included three physiotherapists experienced in the musculoskeletal disorders including LBP, an experienced methodologist in the field of cultural adaptation and validation of health questionnaires, and four translators. Thirty patients with chronic LBP participated in the pilot test of pre-final Persian LSIQ. Patients found no problem with the content and understanding of the items. The expert panel then finalized the Persian LSIQ (LSIQ-P) for psychometric evaluation.

### Psychometric assessment of the final Persian LSIQ

#### Participants

Patients with chronic non-specific LBP (duration for ≥ 3 months), aged ≥ 18 years old who were able to read and write Persian fluently were included, otherwise were excluded.

This study included 100 patients with chronic LBP and 100 neurologically healthy subjects without LBP according to the guideline [23]. Fifty patients with LBP were considered for test–retest reliability.

#### Procedure

The subjects were recruited from May to September 2020 in Iran via the Internet. The online survey was adopted due to the COVID-19 pandemic condition. The link to participate in the study was provided using messages on health and public groups of Telegram and WhatsApp. The message had described the study aims and criteria for participating in the study. Eligible subjects were invited to click on the study link and view the questionnaires. Before participating in the study, the subjects were given information on the study aims and eligibility criteria. If eligible, they were asked to tick the consent box before being allowed to fill up the questionnaires. If unwilling to participate in the study, they were automatically signed out of the page and thus the process was discontinued. Eligible and willing participants were asked to answer demographic questions including gender, age, and duration of LBP. Patients with LBP were requested to provide the E-mail address, if interested, for test–retest phase of the study. Patients completed the LSIQ-P, Persian Functional Rating Index (FRI) [21], and the pain numerical rating scale (NRS) for construct validity [24]. We hypothesized a significant moderate correlation between the LSIQ-P and the Persian FRI as well as NRS. For test–retest reliability, the patients completed the LSIQ-P after 7 days. The healthy individuals completed the Persian LSIQ questionnaire only on the test phase of the study. The questionnaires administered via the Internet have been found to be reliable [25].

### Measures

#### Lumbar spine instability questionnaire

The LSIQ is a 15-item self-reported questionnaire designed to measure the instability of the spine. The LSIQ items ask about the history of trauma/injury, frequency of pain, pain relief on movement, activities associated with pain, need spine movement, duration of symptoms, and fear of moving the spine [13]. The LSIQ total score ranges from 0 to 15 points and higher scores indicate greater spine instability [15].

#### Functional rating index

The FRI is a 10-item, self-reported measure of pain and function developed for assessing disability in patients with LBP or neck pain [26]. The FRI uses a five-point scale to rate each item from “0” (no pain/no dysfunction) to “4” (severe pain/disability). The total score ranges from 0% (no pain/disability) to 100% (severe pain/disability). The FRI has been validated into Persian language in patients with LBP [21] as well as neck pain [27].

#### Numerical rating index

The self-reported NRS was used to quantify the pain intensity from “0” (no pain) to “10” (worst possible pain) [24, 28]. The patients were asked to indicate a number correspondent to their pain intensity. The NRS is a reliable and valid scale for measuring pain intensity [29].

### Statistical analysis

The percentage of patients with a minimum and maximum total score was calculated to determine the floor and ceiling effects; ≥ 15% was indicated significant. The Cronbach’s α was used to determine the internal consistency reliability. The Cronbach’s α of at least 0.7 was considered acceptable. Corrected item-total correlation was used to examine the internal construct validity. The cut-off for Item-total correlation was set at > 0.40 [30, 31]. The intraclass correlation coefficient (ICC_agreement_, two-way random effects model, single measure, and absolute agreement definition) was used to determine the test–retest reliability; the values ≥ 0.7 was considered acceptable. ICC values were interpreted good (> 0.75), moderate (0.75–0.50), and poor *(*< 0.50) [32]. The standard error of measurement (SEM, σ√1-ICC) and the smallest detectable change (SDC, 1.96 × √2 × SEM) were calculated as absolute reliability measures. Pearson/Spearman correlation coefficients were used to determine the construct validity of the LSIQ by associating it to the FRI and NRS. A priori hypothesis was set with positive and moderate correlations between the LSIQ-P and the comparator instruments. The coefficients were interpreted as low (< 0.3), moderate (0.3–0.6) and high (> 0.6) [23, 33]. The independent t test was used to analyze the discriminant validity with patients with LBP and healthy subjects. Mann Whitney U Test was used to analyze the groups of patients with LBP based on the suggested cut-off score of ≥ 9 on LSIQ [15]. It was assumed that the Persian LSIQ is able to discriminate the patients with a higher LSIQ score from those with low score. A principal component analysis with varimax rotation was applied to analyze the factor structure of the LSIQ-P and to determine the possible components of LSIQ-P. The Kaiser–Meyer–Olkin (KMO) of sampling adequacy and the Bartlett's test of sphericity for appropriateness of correlation matrix were calculated. The eigenvalues > 1, the scree plot, and the variance > 10% [34] were applied to determine the number of factors. SPSS software (version 25, SPSS Inc., Chicago, IL) was used to analyze the data.

## Results

A total of 100 patients with LBP (80 women, 20 men) with a mean age of 36.8 years (SD 11.22) and LBP duration of 41.5 (SD 58.7) months participated in the present study. Eighty and eight patients with LBP had ≥ 12 years education. The LSIQ-P total score was not statistically significant between men and women (mean difference: 0.69, t = 0.84, *p* = 0.4).

There was no problem in the translation process of LSIQ into Persian. Patients responded to all items of LSIQ and thus there was no missing data. Patients commented the questions as being clear and understandable.

There were no significant floor and ceiling effects and the range of LSIQ-P scores were well distributed (Table 1).

**Table 1:** Scores of Persian LSIQ, FRI, and NRS (n = 100)

Outcomes	Mean(SD)	Min–Max
Persian LSIQ	9.60 (3.27)	2.0–15.0
Persian FRI	13.80(5.86)	0.0–31.0
NRS	(Median, IQR) 4 (3–5)	–

*LSIQ* lumbar spine instability questionnaire, *FRI* functional rating index,

*NRS* numerical rating scale, *SD* standard deviation, *IQR* interquartile range

Cronbach's alpha was 0.767. Cronbach's alpha, if an item deleted, ranged between 0.741 (question 13) and 0.776 (question 15). The corrected item-total correlation for 8 Items of LSIQ-P were < 0.40 (Table 2) and when deleted the Cronbach's alpha for internal consistency of LSIQ-P (71items) was 0.727 (n = 100).

**Table 2:** Cronbach’s α if item deleted and corrected item-total correlation for Persian LSIQ (n = 100)

LSIQ	Scale mean if item deleted	Scale variance if item deleted	Corrected item-total correlation	Cronbach’s α if item deleted
Q1	9.03	9.039	0.468	0.744
Q2	9.02	9.333	0.366*	0.755
Q3	8.70	10.253	0.178*	0.767
Q4	9.12	9.177	0.413	0.750
Q5	8.86	9.354	0.422	0.749
Q6	8.98	9.131	0.447	0.747
Q7	8.85	9.280	0.459	0.746
Q8	8.74	9.952	0.279*	0.761
Q9	8.72	10.082	0.240*	0.764
Q10	9.10	9.000	0.476	0.743
Q11	8.90	9.424	0.371*	0.754
Q12	8.96	9.291	0.395*	0.752
Q13	8.92	9.044	0.505	0.741
Q14	9.14	9.394	0.339*	0.757
Q15	9.36	10.213	0.106*	0.776

*Item-total correlation < 0.4

Test–retest reliability for the LSIQ-P showed an ICC_agreement_ of 0.78 (95% CI: 0.64–0.87), *p* < 0.001). The absolute reliability measures of SEM and the SDC were calculated 1.53 and 4.24 for the LSIQ-P, respectively.

The Pearson correlation analysis for construct validity demonstrated a significant correlation between the LSIQ-P and the Persian FRI (r = 0.44, *p* < 0.001). The Spearman correlation coefficient between the LSIQ-P and the NRS was 0.30 (*p* = 0.003).

For discriminant validity, the data from the patients in test phase of the study were analyzed with those of healthy subjects (n = 100; 70 female; mean ± SD age 30.7 ± 9.9 years; range 18–60.0; education 87 subjects had ≥ 12 years). Independent t test revealed significant difference of the LSIQ scores between patients with LBP (13.80 ± 5.86) and healthy subjects (2.13 ± 1.85) (t = 19.88, df = 156.58, *p* < 0.001). The differences between patients with LSIQ-P scores ≥ 9 (11.68 ± 2.02) and those with < 9 (6.21 ± 1.68) was statistically significant (Z = -8.41, *p* < 0.001).

**Fig. 1:**
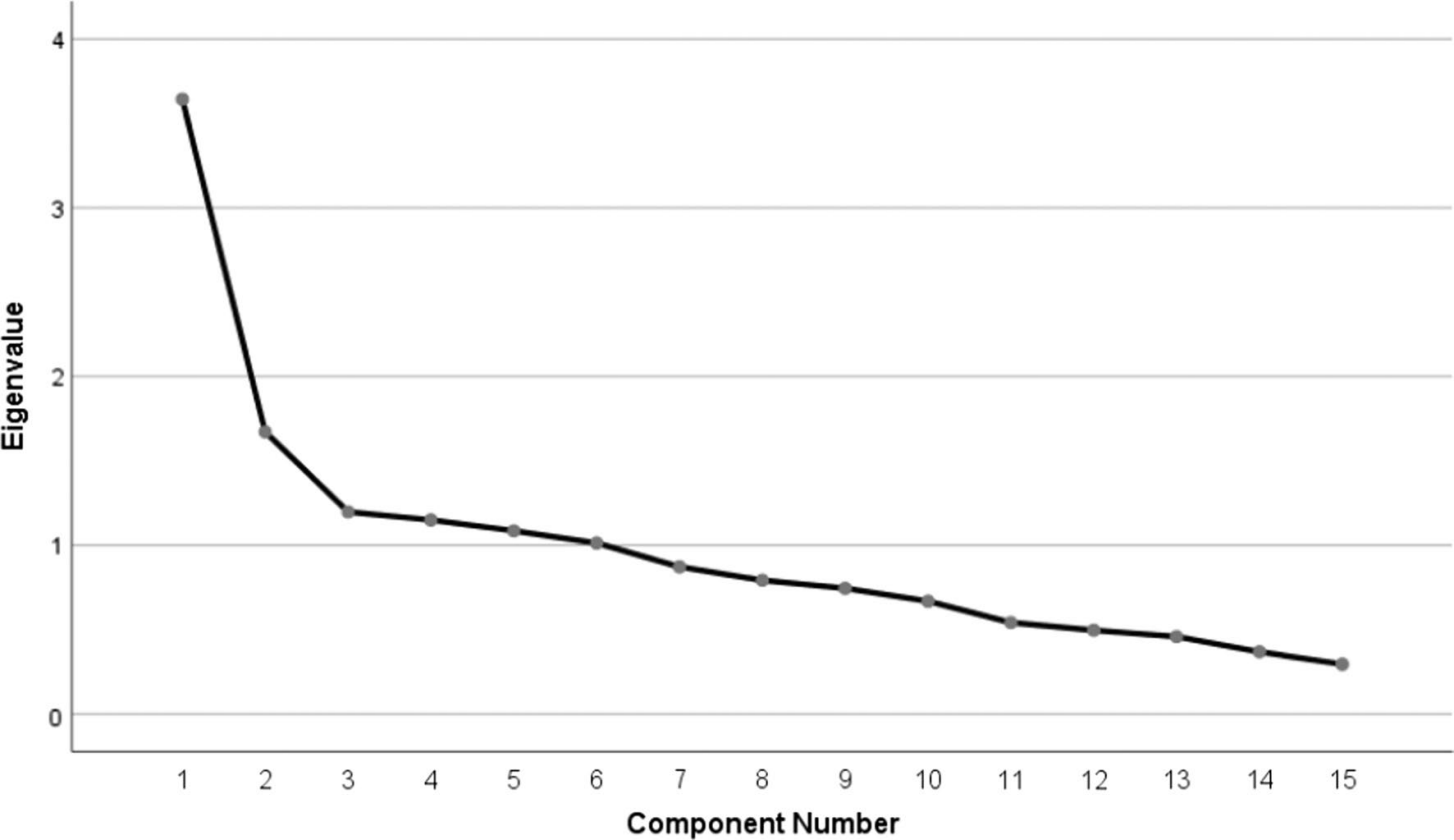
Scree plot of 15-item Persian Lumbar Spine Instability Questionnaire for measuring instability shows a 2-factor solution with the eigenvalues > 1 and variance > 10%

The Kaisere Meyere Olkin (KMO) (KMO = 0.70) and the Bartlett’s test of sphericity (Chi-Square 294.03, df 105, *p* < 0.001) indicated the sampling adequacy and appropriateness of the correlation matrix. Factor analysis extracted 6 components, which explains 65.08% of the total variance. However, the first two factors had the eigenvalues > 1 and variance > 10% explaining 35.43% of the total variance (Table 3, Fig. 1).

**Table 3:** Rotated component matrix with extraction method of Principal Component Analysis and Varimax rotation method

Item	Description	Component					
		1	2	3	4	5	6
Q1	I feel like my back is going to “give way” or “give out” on me		0.719				
Q2	I feel the need to frequently pop my back			0.659			
Q3	I have frequent bouts or episodes of symptoms						0.912
Q4	In the past my back catches or locks when I twist or bend my spine		0.478			− 0.529	
Q5	I experience pain when I change positions (e.g., sit to stand or stand to sit)			0.465		− 0.413	
Q6	When I bend forward it hurts, but returning to standing is usually worse	0.659		0.449			
Q7	My pain increases with quick, unexpected, or mild movements	0.778					
Q8	I have difficulty sitting without a back support (such as a chair) and feel better with a supportive backrest				0.856		
Q9	My pain is usually worse with prolonged or static positions					0.787	
Q10	It seems like my condition is getting worse over time (e.g., shorter intervals between bouts)		0.491	0.522			
Q11	I have had this problem for a long time		0.426			0.433	
Q12	I get temporary pain relief with a back brace or corset	0.595					
Q13	I have many occasions when I get muscle spasms in the back	0.568	0.447				
Q14	I am sometimes fearful to move because of my pain		0.728				
Q15	I have had a back injury or trauma in the past			0.648			
Eigenvalue		3.643	1.672	1.198	1.150	1.086	1.013
% of Variance		24.285	11.147	7.985	7.667	7.238	6.754

Exploratory factor analysis with varimax rotation and deleting 8 items with item-total correlation < 0.4 extracted one component with items 1, 4–7, 10, and 13 that explained 38.13% of variance (eigenvalues = 2.67). Component matrix of remaining 7 items indicated factor loadings between 0.552 (item 10) and 0.664 (item 13). Factor loadings for items 4–7 were 0.626, 0.622, 0.647, 0.628, respectively, and factor loading for item 10 was 0.552.

## Discussion

This study translated and culturally adapted the LSIQ into Persian language and provided a reliable and valid measure in line with a study that used the original English version [16], and with recent studies of Swedish [20], Thai [17, 18] and Brazilian-Portuguese [19]. The present study found sound psychometric properties on the various reliability and validity metrics of LSIQ-P supporting it as a useful questionnaire for assessing patients with LBP.

Patients responded to all items of the LSIQ-P. The responding to all items indicates that the patients were able to understand and complete the LSIQ-P easily. This finding reflects the applicability and feasibility of the LSIQ-P. This is in line with the Original English and translated versions of the LSIQ [16, 17, 19, 20].

Floor or ceiling effects were not detected for the LSIQ-P. This finding is in line with previous studies on the original and adapted versions of LSIQ [15, 19, 20]. The lack of floor or ceiling effects in the current study may reflects the LSIQ-P responsiveness. Nevertheless, a study on the responsiveness of LSIQ-P is required in a clinical context with interventions to confirm its responsiveness.

In the present study, the internal consistency reliability was 0.767 and exceeded the acceptable cut-off score of 0.7 in line with the Brazilian-Portuguese version [19] that reported good internal consistency reliability (α = 0.790). The corrected item-total correlation in the present study did not exceed the cut-off value of 0.40 [31] for all LSIQ-P items. The corrected item-total correlation to examine the internal structure of a test defines the association between an item with the total score. Low corrected item-total correlation found in this study suggests that the consistency between an item and the other items in the LSIQ-P was not adequate to consider it as a one-dimensional measure. The previous studies with the English LSIQ found Cronbach’s alpha which were lower than acceptable boundary value (0.69, [15]; 0.63, [16]). Moreover, the alpha for the Swedish version did not reach the recommended threshold (α = 0.64, [20]). Altogether, these findings may indicate that the construct of the LSIQ is not homogenous and is comprised of different latent constructs. The LSIQ, in fact, includes items asking general questions on LBP as well as items on lumbar clinical instability. This implies an uncertainty in unidimensionality of the LSIQ in measuring only the construct of clinical instability.

Test–retest reliability for the LSIQ-P total score was excellent (ICC 0.78) [35]. This finding is in line with those reported for the Swedish (ICC = 0.94, [20]), original English (ICC = 0.84) [16], and Brazilian-Portuguese (ICC = 0.75) [19]. Test–retest reliability is an important psychometric property of a questionnaire for use in the clinical and research settings as it indicates the questionnaire stability over time. We calculated the ICC_agreement_ for test–retest reliability because it takes the systematic error into account [36].

The SEM and SDC were computed as the agreement measures of reliability. Agreement parameters are important for clinical interpretation of questionnaire scores as they can be applied in all situations used for assessment purposes [37]. The values of SEM and SDC obtained for an instrument is useful in the clinical context as it will help the clinicians to find out how much changes is real after an intervention beyond the measurement error. The low SEM obtained for the LSIQ-P indicates that it is a reliable as well as sensitive for revealing real changes after treatment. The SDC is a better measure than the SEM as it enables the clinicians to judge whether the changes on the LSIQ-P measured for an individual patient pre and post treatment is real. According to the SDC value of 4.24 calculated for LSIQ-P, a total score at least 5 points is needed to be interpreted as real change. Change score of less than ≤ 4.24 on the LSIQ-P indicates a measurement error that is not clinically valuable.

This study, as priority hypothesized, found a moderate correlation between the LSIQ-P and the Persian FRI or the NRS that confirms the construct validity of the LSIQ-P. It is in line with findings from English as well as other language versions of LSIQ that also reported identical moderate correlation coefficient [16, 19, 20].

Discriminant validity of the LSIQ-P was assessed by comparing the scores of patients with LBP and those of healthy subjects. The LSIQ scores of patients with LBP were significantly worse than those of healthy subjects that support the discriminant validity of the LSIQ-P in distinguishing patients with LBP from healthy subjects.

The LSIQ is hypothesized to distinguish patients with LBP who achieve better outcomes with motor control exercise (≥ 9 points) and those who respond to graded activity (< 9 points) [15, 38]. The LSIQ-P demonstrated significant differences between LBP patients with high clinical instability (scores 9 ≥) and those with low instability (scores < 9) that suggests its known-group validity. This finding is in line with the Swedish version [20] and with previous validity studies of English LSIQ [15, 16]. However, a further study with the English LSIQ concluded that more items may be required to enhance its ability to discriminate individuals with high instability from those with low instability [38]. The Brazilian-Portuguez version of LSIQ is not evaluated for discriminant validity [19].

The factor analysis of the LSIQ-P showed that the LSIQ may not be a single factor questionnaire. The corrected item-total correlation analysis revealed that 8 items of the LSIQ-P may be redundant, and it was only with removal of 8 items that a single factor achieved. This finding indicates that 8 items of the LSIQ evaluates different constructs confirming the multidimensionality of the LSIQ. While previous studies applied Rasch analysis and reported that the LSIQ is unidimensional measure [16, 38], authors found low Person Separation Index value [38] and several items that were biased by factors other than the instability construct [16]. We did not apply a Rasch analysis to investigate the dimensionality of the LSIQ-P. Future investigation is required to conduct a Rasch analysis of the LSIQ-P to determine the dimensionality. The other language versions of the LSIQ did not evaluate the factor analysis and questionnaire dimensionality.

There are limitations in the present study. First, there is a lack of measure for lumbar spine instability for construct and criterion validity evaluation. We used the pain and function measures of FRI and NRS. Recently, validation investigations of LSIQ used the Roland Morris Disability Questionnaire and the NRS for construct validity [16, 19, 20]. Second, the Rasch analysis was not performed in this study. However, we used both exploratory factor analysis and item-total correlation that revealed the LSIQ-P is multidimensional and several items being redundant in line with a previous study [16]. Third, responsiveness study of LSIQ-P is required to determine the threshold value as clinically important change. Fourth, this study recruited subjects online via internet. Thus, we were not able to verify the participants’ back pain and lumbar instability. However, people from various cities in Iran participated in this study and responded to all items of the questionnaire. This indicates the generalizability of the study findings.

## Conclusion

The present study provided a reliable and valid metrics for LSIQ-P probably derived from those with LBP and clinical lumbar spine instability. The LSIQ-P may be used for making a clinical assessment of the lumbar spine instability for Persian language context. Factor structure of the LSIQ-P showed that the 8 items of the LSIQ-P are candidates for removal. Future studies are required to elucidate the internal structure and underlying construct of the LSIQ-P.

## Data Availability

The datasets used and analyzed in this study are available from the corresponding author (NNA) on reasonable request.

## Abbreviations

LSIQ: Lumbar spine instability questionnaire
LBP: Low back pain
FRI: Functional rating index
NRS: Numeric rating scale
SEM: Standard error of measurement
SDC: Smallest detectable change
ICC: Intraclass correlation coefficient
